# Supplementary material for: Remote assessment of pelvic kinematics during single leg squat using smartphone sensors: Between-day reliability and identification of acute changes in motor performance
Source: PLoS One. 2023 Nov 22;18(11):e0288760. doi: 10.1371/journal.pone.0288760 (PMC10664960; doi:10.1371/journal.pone.0288760)
Supplement: Attachment — Submitted filename: Response to Reviewers.docx [file pone.0288760.s003.docx]

Reviewer 1

The feasibility of using smartphone sensors for biomechanical assessment of pelvic kinematics during a single leg squat (SLS) in ecological settings was investigated. Thirty-three healthy young adults participated remotely on two different days using their own smartphones placed on the lumbosacral region. Pelvic orientation and acceleration data were collected during SLS sets and an endurance task. The reliability of the measurements was assessed using Intraclass Correlation Coefficient (ICC2,k), Standard Error of Measurement, and Minimal Detectable Change. Results showed good to excellent reliability (ICC2,k ≥ 0.79) for pelvic orientation and frequency features of acceleration signals. Changes in pelvic kinematics during the endurance task were identified, including larger contralateral pelvic drop and rotation. These findings indicate that smartphones can provide reliable measurements of pelvic kinematics remotely during SLS, and also detect changes in motor control during an endurance task.

I have completed the review of the manuscript in question, and overall, I find the study to be well-written with an interesting practical application. Despite the use of "commercial" equipment, the methodological approach is robust. The results are clearly presented and comprehensive, as are the accompanying figures. The discussion is well-supported by the results.

I have only one point that I believe the authors should address: Was the calculation of the sample size performed? The term "convenient sample" is somewhat ambiguous, and it would be beneficial for the authors to clarify this aspect in the text, if such calculations were indeed conducted.

We thank the reviewer for the positive feedback about our study.

Regarding the comment on the sample size, we expanded the section explaining that we adopted current statistical methods relying on the interclass correlation coefficients to identify the required sample size.

Line 83: “*The sample size was calculated using an online application (17) and the following parameters: alpha = 0.05, power = 80%, minimum acceptable ICC = 0.7, expected ICC = 0.9 (based on other reliability studies of smartphone sensors (13). The calculation resulted in a minimum sample size of 23 participants. However, to account for a potential larger-than-usual dropout rate due to the remote nature of the study, we increased the number of participants to 33*.”

In addition to the revisions requested by the reviewer, we have also slightly revised the text for grammar and clarity (track-changed).

13. Keogh JWL, Cox A, Anderson S, Liew B, Olsen A, Schram B, et al. Reliability and validity of clinically accessible smartphone applications to measure joint range of motion: A systematic review. Müller J, editor. PLOS ONE. 2019 May 8;14(5):e0215806.

17. Arifin WN. A Web-based Sample Size Calculator for Reliability Studies. Educ Med J. 2018 Sep 28;10(3):67–76.
